# Mapping the Spatiotemporal Distribution of Bovine Rabies in Colombia, 2005–2019

**DOI:** 10.3390/tropicalmed7120406

**Published:** 2022-11-29

**Authors:** D. Katterine Bonilla-Aldana, S. Daniela Jimenez-Diaz, Joshuan J. Barboza, Alfonso J. Rodriguez-Morales

**Affiliations:** 1 Research Unit, Universidad Continental, Huancayo 12000, Peru; 2 Faculty of Veterinary Medicine, Fundación Universitaria Autónoma de las Américas, Pereira 660003, Risaralda, Colombia; 3 Vicerrectorado de Investigación, Universidad Norbert Wiener, Lima 15046, Peru; 4 Faculty of Health Sciences, Universidad Científica del Sur, Lima 15024, Peru; 5 Grupo de Investigación Biomedicina, Faculty of Medicine, Fundación Universitaria Autónoma de las Américas, Pereira 660003, Risaralda, Colombia; 6 Institución Universitaria Visión de las Américas, Pereira 660003, Risaralda, Colombia; 7 Gilbert and Rose-Marie Chagoury School of Medicine, Lebanese American University, Beirut P.O. Box 13-5053, Lebanon

**Keywords:** bovines, *Lyssavirus*, bats, rabies, public health

## Abstract

Introduction: Rabies is caused by a virus belonging to the genus *Lyssavirus* and family Rhabdoviridae, which can infect any mammal including humans. Hematophagous, fructivorous, and insectivorous bats have become the main reservoir of sylvatic rabies in Latin America. In the sylvatic cycle, hematophagous bats are usually the main reservoir. In contrast, dogs and cats fulfil this critical role in the urban cycle. However, in rural areas, the most affected animals are bovines. They show clinical signs such as behavioural changes, hypersalivation, muscle tremors, spasms caused by extensive damage to the central nervous system, and death from respiratory paralysis. Objective: To describe the spatiotemporal distribution of bovine rabies in Colombia from 2005 to 2019. Methods: Retrospective cross-sectional descriptive observational study, based on the monthly reports of the Colombian Agricultural Institute (ICA) on the surveillance of bovine rabies in Colombia from 2005 to 2019, retrieved from its official website. The data were converted to databases in Microsoft Access 365^®^. Multiple epidemiological maps were developed with the GIS software Kosmo RC1^®^ 3.0 coupled to the shape files (.shp) of all the country’s municipalities. Results: During the study period, 4888 cases of rabies were confirmed in cattle, ranging from a peak of 542 cases (11.1%) in 2014 to 43 in 2019 (0.88%). From 2014 to 2019, there has been a significant reduction in the annual national number of cases (r^2^ = 0.9509, *p* < 0.05). In 2019, 32.6% of the cases occurred in January, and 48.8% occurred in the department of Sucre. In 2009, the maximum number of spatial clusters (13) occurred in the Orinoquia region, where other clusters were also identified in 2005, 2006 and 2008. In 2018, 98 outbreaks were identified that led to the death of cattle and other animals, 28.6% of them in the department of Sucre. In the first half of 2019, of 38 outbreaks, 55.2% were identified in Sucre. Conclusions: It is necessary to review the current national program for the prevention and control of rabies in cattle, incorporating concepts from the ecology of bats, as well as the prediction of contagion waves of geographical and temporal spread in the context of the OneHealth Approach. Sylvatic rabies remains a threat in Colombia that requires further study.

## 1. Introduction

Rabies is a zoonotic and deadly virus with significant tropism for the central nervous system (CNS). It has affected the world for more than two thousand years [1,2]. The disease is caused by a virus belonging to the genus *Lyssavirus*. It is part of the Rhabdoviridae family; it is an enveloped RNA-virus with bullet-shaped morphology and composed of a single non-segmented chain. The rabies virus infects all warm-blooded animals, and the most affected are mammals, including bovines, sheep, goats, canines, felines, and humans [3,4,5]. Naturally, the control of rabies in domestic, farm and sylvatic animals is highly relevant from an integrated perspective [6]. Rabies is a critical public health threat, so the World Health Organization (WHO), the World Organization for Animal Health (OIE), the Food and Agriculture Organization (FAO), and the Global Alliance for Rabies Control (GARC) all agreed on The Global Strategic Plan to End Human Deaths from Dog-Mediated Rabies by 2030. It has been estimated that more than 99% of human rabies cases are associated with dog bites; unfortunately, most occur in children. The role of wildlife remains widely unknown in terms of a constant potential reservoir as a source of reintroduction or spillover; thus it remains neglected in most rabies control programs [7].

The rabies virus has two disease cycles: sylvatic rabies and urban rabies. In sylvatic rabies, the blood-sucking bats, commonly present in Latin America, play the most critical role. On the other hand, for rabies of urban origin, domestic dogs and cats play an essential role in the transmission and sometimes act as intermediate hosts to infect humans. Cats can become rabid and infect humans, but are not a reservoir species [8]. In addition, the virus can be transmitted by bites and scratches between animals of the same or different species [9,10]. For example, cattle are exposed to bat bites. In multiple countries, bat species, such as *Desmodus rotundus*, are essential in rabies transmission and the cause of outbreaks in cattle and deaths in humans, especially considering the advance of humans into previously preserved ecosystems, also introducing livestock. There seems to be a correlation between the impact of anthropic changes to the environment, mainly for the expansion of pasture for cattle, and the outbreaks of bovine rabies, as has been recently demonstrated in the state of São Paulo, Brazil [11].

The rabies virus has an incubation period of 25 to 150 days, after which clinical manifestations begin. Apathy, muscle tremors, pain at the bite site, hyperexcitability, spasms, frequent vocalisations, excessive frothy salivation, bristling hair, and subsequent death in a high proportion of cases, due to paralysis of the respiratory system, are observed in infected animals, including cattle [9,12].

Although multiple studies have been conducted on rabies in humans and domestic animals, less is known about wild [13] and farm animals, including cattle [14]. A previous study in Colombia found that the departments of Cesar, Córdoba, Antioquia, Choco, La Guajira, Arauca, Santander and Casanare presented a higher number of sylvatic rabies foci and concluded that they are those with the highest risk of transmission of the disease [4]. Other studies have been carried out on the geographical distribution of the risk of sylvatic rabies, and have evaluated the factors associated with its incidence in Colombia; it was determined that municipalities such as Montería, Valledupar, Riohacha, Aguachica, Unguía, Acandí, Río de Oro, Tibú, Sahagún and San Onofre are among those with a high risk of presenting rabies of sylvatic origin [4,15]. However, such studies did not include bovine populations.

The virus has a case fatality rate near 100%. Most of the infected animals die from this disease, although, for example, the small Indian mongoose can survive rabies infection as many wild-caught animals have high levels of circulating virus-neutralising antibodies [16,17]. The introduction of livestock from different countries and the modification of the reservoirs’ natural habitats have increased the size and the number of colonies of vampire bats [18]. This causes multiple economic losses for farmers whose daily sustenance is livestock production; currently, Colombia has implemented activities for the control of this disease, including vaccination, especially for dogs, vector control, monitoring of outbreaks and characterisation of areas of risk [19], but there are not updated studies on how the spatiotemporal distribution of this disease has developed in recent years. The vaccination programs should be enhanced and should consider the importance of immunisation for disease control [20,21]. Living closer to urban areas causes animals to be bitten and infected, since bats commonly inhabit buildings, roofs, wall crevices, and hollow logs [4,22].

In the present study the objective was to describe the spatiotemporal distribution of bovine rabies in Colombia from 2005 to 2019.

## 2. Materials and Methods

### 2.1. Type of Study

A quantitative, observational, descriptive, cross-sectional retrospective study on the incidence of rabies in cattle (bovine) was carried out. The incidence rate was estimated per year by departments of Colombia, and epidemiological maps of the diseases were developed between 2005 and 2019.

### 2.2. Data Source, GIS-Mapping and Statistical Analyses

In the study of secondary sources, the bulletins of the Colombian Agricultural Institute (ICA) were the main ones used. They contain sociodemographic characteristics and the diagnosis of the disease. In addition, the total number of cases and their regional locations were taken, and the geographical characterisation of the incidence of rabies in bovines began. A case was defined as any bovine with suspicion of rabies, with or without clinical findings, which was confirmed by laboratory (PCR).

For the respective geographical characterisation, the free access software Kosmo 3.1 was used, which has tools preloaded from the geographical point of view; for this research, we worked with Oeste Bogotá (West Bogotá) (Magna SIRGAS) as a reference system (EPSG 21896). The mapping was carried out (at a scale of 1:1,365,207) and two types of layers were used; the first layer, the departments, had colours of greater or lesser intensity added to it as defined by the second layer, of epidemiological data. This was classified by ranges established by quartiles, which allow differentiation of the areas with a higher incidence of the disease from those with a lower incidence (according to the technique of quartiles/quintiles of cases/100,000 inhabitants). Using the free software SaTScan, ver. 10.1, we performed a cluster analysis for some years when municipality data were available in addition to departments. A Poisson model was used to explore the areas with the highest risk in each study year. The maximum size of the cluster was set at 50% of the bovine population of risk, and we represented those with a value of *p* < 0.05.

Descriptive analyses were performed on Stata IC^®^ version 14. The summary of cases per year and department, and the median number of cases per month and year for specific periods were calculated. As part of the time-series analyses, linear regression for the trend from 2014 to 2019 for the country, as well as for the departments, was calculated with a 95% confidence level (*p* < 0.05) estimating the coefficient of determination (r^2^). Annual incidence rates were calculated as the number of cases of rabies in bovines divided by the estimated population of bovine animals per 100,000 bovines.

### 2.3. Population and Sample

Colombia’s total bovine population for 2019 was estimated at 27,234,027 [23]. The sample was taken from the census from the ICA databases, from which registered data were obtained from cases of diagnosis of rabies in bovines in Colombia between 2005 and 2019. For 2016 to 2019, the annual number of bovines per department was obtained. For that period, the incidence rate of bovine rabies was estimated by calculating the number of cases per 100,000 bovines per department per year.

## 3. Results

During the study period, 4888 cases of rabies in bovines were confirmed in the different departments of Colombia.

**Figure 1:**
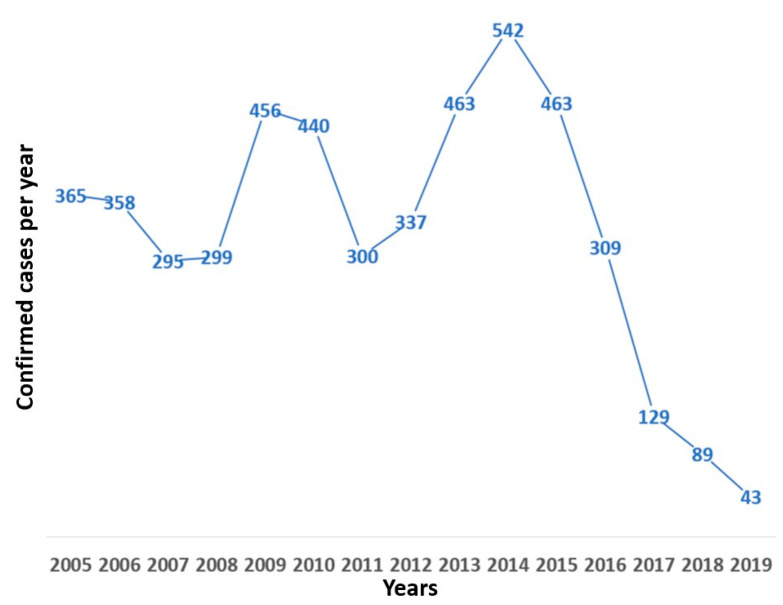
Bovine rabies, cases per year, Colombia, 2005–2019.

**Figure 2:**
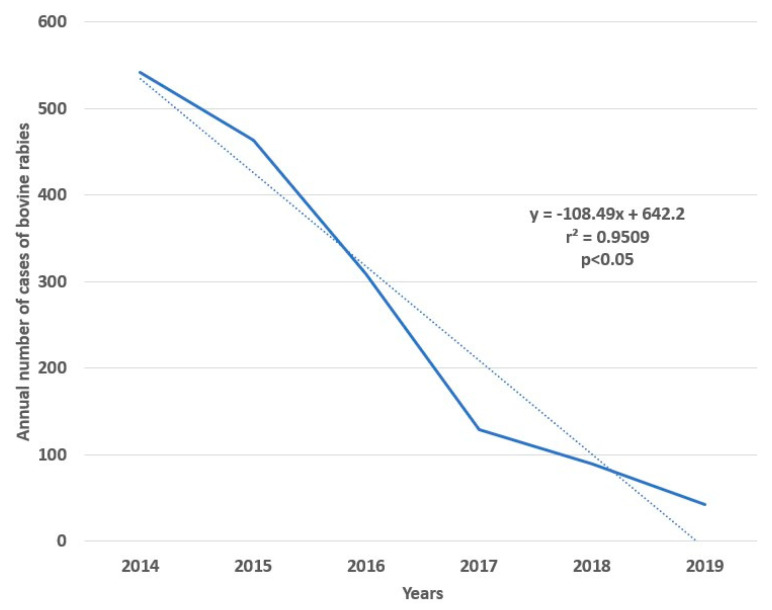
Case trend from 2014 to 2019, bovine rabies, Colombia.

Regarding its temporal distribution, the number of cases varied from a maximum of 542 cases (11.1% of the total) in 2014 to a minimum of 43 in 2019 (0.88%) (Figure 1). A linear regression analysing from 2014 to 2019, showed a statistically significant reduction in the annual national number of cases (r^2^ = 0.9509, *p* = 0.0009, F = 77.52, root MSE = 51.545) (Figure 2).

In four departments, Cesar, Chocó, Norte de Santander, and Magdalena, a significant variation, and reduction, in the number of cases of bovine rabies was observed between 2014 and 2019 (Table 1).

**Figure 3:**
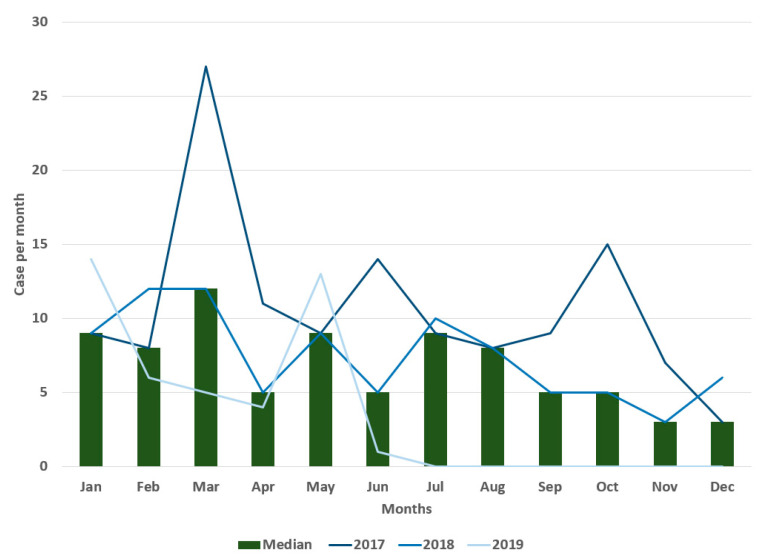
Number of cases and median by months from 2017 to 2019, bovine rabies, Colombia.

In 2019, 32.6% of the cases occurred in January. In 2019, 48.8% occurred in the department of Sucre. For the period 2017–2019, the median number of cases was highest in March (Figure 3) but not significantly higher than other months (*p* > 0.05).

**Figure 4:**
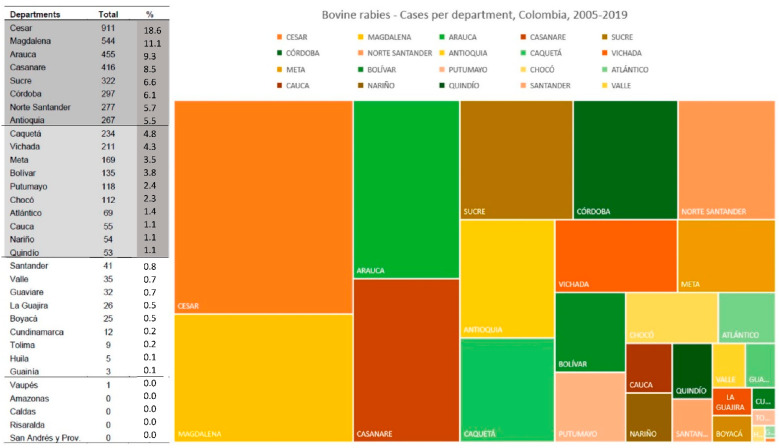
Distribution of bovine rabies cases by departments, Colombia, 2005–2019.

The department with the highest number of reported cases was Cesar, with 911 (18.6%), followed by Magdalena with 544 (11.1%), Arauca (455, 9.3%), Casanare (416, 8.5%), and Sucre (322, 6.6%), among others (Figure 4). Peaks were observed in these departments, especially in 2005, 2009–2010, 2012 and 2014 (Figure 4).

**Figure 5:**
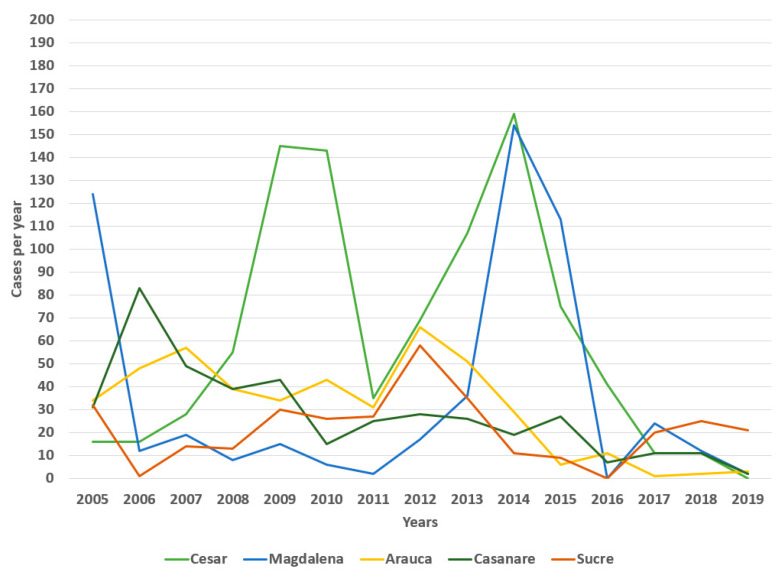
Departments with the highest bovine rabies cases, Colombia, 2005–2019.

In 2009, with municipality data, spatial distribution was analysed. The maximum number of spatial clusters (13) occurred in the Orinoquia region, where the department of Vichada stands out (Figure 5), and where other clusters were also identified in 2005, 2006 and 2008.

**Figure 6:**
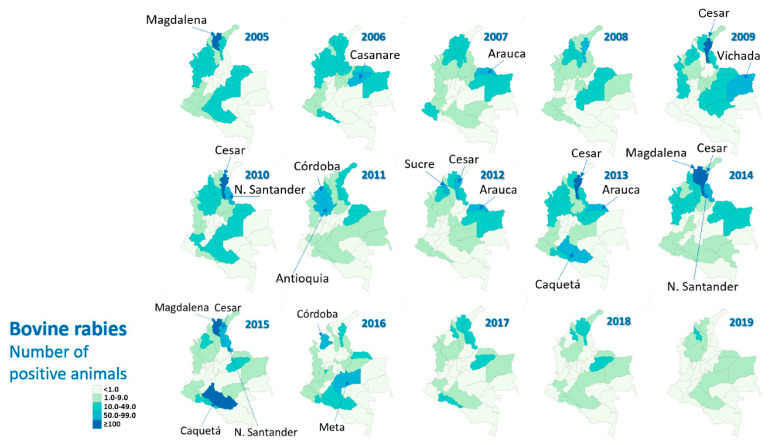
Geographical distribution of bovine rabies by department, Colombia, 2005–2019.

In 2015, the most important departments for bovine rabies were Magdalena, Cesar, Caquetá and Norte de Santander (Figure 6), while in 2016, they were Meta and Córdoba (Figure 6).

In 2018, 98 outbreaks were identified that led to the death of cattle and other animals, 28.6% of them in the department of Sucre. In the first semester of 2019, of 38 outbreaks, 55.2% were identified in Sucre. No cases of human rabies have been reported in Sucre during those years. As seen in Figure 6, in recent years, bovine rabies has decreased in the country, specifically in the departments with the highest incidence.

For 2016–2019, the highest incidence rates (cases per 100,000 animals) were observed in 2016, with 1.362 cases per 100,000 animals (Table 2). In 2016, the department with the highest incidence rate was Quindio (58.688 cases per 100,000 animals). However, the national rates decreased in the following years to 0.158 cases per 100,000 animals (1.58 cases per 1,000,000 animals) in 2019. For 2017 and 2018, the department with the highest incidence rate was Cesar (7.106 and 5.504 cases per 100,000 animals, respectively). However, Quindio and Cesar later decreased those incidences. In 2019, although there were just three cases, the department of Bolivar increased its incidence rate to 8.498 cases per 100,000 animals (Table 2).

## 4. Discussion

As seen in the results, the number of cases reported in the study period continues to be significant, as there should be no cases of bovine rabies. Data could correspond to underreporting, although it decreased during the last five years. If those data were accurate, we would expect that Colombia would shortly be able to eradicate rabies in bovines. Nevertheless, enhanced surveillance, including active searches for foci, and sentinel surveillance by animal health authorities, as well as more education for the population and the academic sector, mainly oriented to farmers and producers, are needed. Awareness about the health and economic impacts of bovine rabies should also be raised.

Rabies in cattle represents a considerable public health problem and also affects production and subsequent economic impact. In 2013, a study from Colombia was published analysing the geographical distribution of sylvatic rabies from 1982 to 2010. For that period, 2330 outbreaks were observed, distributed in 359 (31.8%) of the 1128 existing municipalities in the country. The researchers classified 144 municipalities at high risk. The highest incidence rates were concentrated in the municipalities of Montería (Córdoba), Valledupar (Cesar), Riohacha, Aguachica (Cesar), Unguía, Acandí, Río de Oro, Tibú, Sahagún and San Onofre [4]. As can be seen in our results, the departments of Córdoba and Cesar continued to present a critical situation with rabies, in this case in cattle, between 2010 and 2019, in addition to other departments that we assessed in this study.

The results of rabies distribution in cattle in 2005–2019 in, for example, departments such as Córdoba, in the Caribbean region of Colombia, may be related to the distribution of rabies in bats, which have recently been reported in studies that have found species such as *Artibeus lituratus* and *Artibeus planirostris*, with the presence of rabies virus, confirmed by molecular analysis and genome sequencing [24]. On the other hand, three urban rabies outbreaks have been reported in Colombia during the last two decades, one of which occurred in the Caribbean Region (northern Colombia), while the other two occurred almost simultaneously in Arauca (eastern Colombia) and in the Central Region. To derive the phylogenetic relationships among the rabies viruses isolated in three areas, 902 nt cDNA fragments encoding the cytoplasmic domain of protein G and a fragment of protein L were obtained by RT-PCR. Phylogenetic analysis showed three distinct groups of viruses in the study sample. The third group isolated the virus from two insectivorous bats, three domestic dogs, and one human. According to the sequence analysis, the isolates correspond in this third group to bats’ variants of the rabies virus. That suggests that the rabies in Colombian dogs and humans was first associated with bats, showing an unsuspected vector that threatens animal and public health [25]. In another study, outbreaks of human rabies transmitted by hematophagous bats were confirmed in the municipalities of Alto Baudó and Bajo Baudó, department of Chocó, Colombia, in 2004–2005 [26].

The department of Magdalena was recorded as one of the most affected departments during 2005–2006. At the beginning of the study, that department presented a significant number of cases, consistent with epidemiological reports published in 2007, which shows that this department presented cases of animal rabies in 2005. Twelve cases were reported in 2006, with two cases of human rabies in the same year caused by the bite of a Dalmatian dog of approximately two months. The first human case in 2006 was a young man, 29 years old, who was bitten on the upper lip on 26 September. He finally died with CNS compromise on 7 November 2006. The second human case was a 14-year-old adolescent who was bitten on his left leg on 11 November, when the canine was prevented from entering his home. He died on 25 November 2006. It was found that the infected animal had bitten eight more people [27].

In epidemiological studies of canine rabies, socioeconomic conditions are associated with its occurrence. In Colombia, a study showed that during the years 1976–2006, 2006 registered the highest number of cases of rabies in bovines [28]; whereas when making a comparison with our results, we observed that although this year presented a significant number of cases, it was not the most relevant year. After 2006, the cases of rabies in bovines have shown an increasing trend that is observed from the year 2013, and which began to decrease after 2015.

Other studies conducted in 2001–2011 analysed sylvatic rabies and its role in the number of bovine outbreaks, indicating that the most affected departments were Antioquia, Arauca, Casanare, Cesar and Córdoba [19]. Furthermore, the departments of Sucre, with its highest peak in 2012, and Magdalena, with its maximum rise in 2014, were significantly affected.

The distribution by years of cases of rabies in bovines (Figure 1) showed that during 2010 a significant number of cases of rabies were presented, consistent with case reports published in the same year [29,30,31]. For example, in the department of Santander, two cases of human rabies caused by the bite of a bat, in a 43-year-old adult and a 14-year-old adolescent who showed post-exposure clinical signs and finally died in a health centre, were reported that year [32].

Studies on the epidemiological profile of rabies in Colombia (2007–2017) showed that in 2011 the department of Casanare was the territory with the second-highest reports of urban rabies, followed by Tolima, and was the highest in 2012 with an incidence of 36.1 cases per 10,000 pop. [33]. That is consistent with the findings of bovine rabies in the current study (Figure 6), where it was observed that the department of Casanare showed a constant trend in the number of cases maintained for some years.

Epidemiological descriptions of human and animal rabies in Colombia made in 2008–2013 also showed findings consistent with this study. The departments of Cesar, Casanare, Arauca, Sucre and Córdoba were the territories with the highest incidence of cases during the study period. Magdalena registered 10 cases of urban rabies, especially during the years 2012 and 2013 [34], consistent with the increase in the number of cases (Figure 5); Cesar presented a significant peak in 2009 and Sucre reached its maximum height in the year 2012.

Spatiotemporal analysis of bovine rabies in Colombia revealed that the highest number of cases occurred in 2014, which represents an evident relationship with our results of the distribution of years by rabies cases (Figure 3), where it is observed that the year was the maximum peak during the study period (2005–2019) [28,34].

Chiropterans continue to be an essential source of contagion of the rabies virus for multiple species, including cattle, with a great connection to registered cases of bovine and human rabies. Despite that, proper surveillance for rabies in bats is not performed in Colombia or many other countries in Latin America. That is key, considering the relationship between bat and bovine rabies [2,11,14,31]. The departments of Antioquia, Arauca, Casanare, Cesar, Magdalena and Córdoba continue to be the first places in the territories with the highest number of rabies cases, which in turn has represented multiple public health problems when the virus comes into contact with urban reservoirs such as dogs and cats that do not have an adequate vaccination plan to prevents the virus from reaching a susceptible host. Therefore, although there have not been a large number of human rabies cases in Colombia, it is of great importance to carry out epidemiological surveillance in the departments above, which report the highest incidence, to help reduce the death of human beings due to this lethal virus, mainly because is still causing human disease in the country [35,36]. Geographical information systems are also valuable for mapping rabies in different populations, as shown in this study [37].

## 5. Conclusions

Since sylvatic rabies continues to be an important source of infection for different animals, and human cases can also occur, its surveillance and control should be strengthened and evaluated based on the present results and the areas of most significant importance in cases should be assessed. It is necessary to review the current national program for the prevention and control of rabies in livestock, incorporating concepts from the ecology of bats, as well as for the prediction of contagion waves of geographical and temporal spread, in the context of a OneHealth approach [38,39,40,41]. Additionally, vaccination is vital for control programs to prevent rabies in livestock and should be increased [42,43]. It is essential to generate greater attention and epidemiological surveillance in the departments that continue to provide a continuous incidence in the number of cases and that, even with time, continue to be in the first territories most affected by bovine rabies, which represents economic losses for the local ranchers and the country [44,45]. It is essential to work with ranchers on ecological importance and environmental conservation, as well as in preserving native forests, which are the natural habitat of the hematophagous bat [46], which would help to reduce the reservoir–host interaction and decrease the number of cases. Given that sylvatic rabies continues to be a threat to Colombia, and is registered as an endemic disease that represents risks to public health because it is zoonotic, highly fatal and can affect all mammals, it is of great importance to carry out more studies on its distribution, risk factors and incidence.

## Figures and Tables

**Table 1:** Variation in bovine rabies cases by department, Colombia, 2014-2019, assessed by linear regression models.

Department	*p*-Value	r^2^	Department	*p*-Value	r^2^
Cesar	**0.0137**	0.8151	Caqueta	0.4111	-
Choco	**0.0189**	0.7844	Cordoba	0.6017	-
Norte de Santander	**0.0329**	0.7192	Tolima	0.6803	-
Magdalena	**0.0351**	0.7105	Boyaca	0.8047	-
La Guajira	0.0595	-	Quindio	0.8047	-
Casanare	0.0708	-	Valle del Cauca	0.8047	-
Arauca	0.0752	-	Meta	0.8322	-
Guaviare	0.0963	-	Cauca	0.9443	-
Sucre	0.1405	-	Amazonas	N/A	-
Bolivar	0.1443	-	Atlantico	N/A	-
Santander	0.1583	-	Caldas	N/A	-
Vaupes	0.1583	-	Cundinamarca	N/A	-
Vichada	0.1894	-	Guainia	N/A	-
Antioquia	0.2857	-	Huila	N/A	-
Nariño	0.3528	-	Risaralda	N/A	-
Putumayo	0.3893	-	San Andres	N/A	-

r^2^ = coefficient of determination. Bold, significant values.

**Table 2:** Bovine rabies cases, bovine population and bovine rabies annual incidence per departments, Colombia, 2016–2019.

	Cases per Year				Population per Year				Annual Incidence (Cases per 100,000 Animals)			
Department	2016	2017	2018	2019	2016	2017	2018	2019	2016	2017	2018	2019
Quindio	48	0	0	0	81,788	76,814	83,425	306,918	58.688	0.000	0.000	0.000
Cesar	41	11	11	0	152,228	154,796	199,854	315,597	26.933	7.106	5.504	0.000
Putumayo	38	12	2	0	197,611	209,006	255,912	482,233	19.230	5.741	0.782	0.000
Cordoba	52	0	0	1	1,256,535	1,146,137	1,422,452	166,411	4.138	0.000	0.000	0.601
Meta	54	0	1	1	1,660,147	1,734,106	1,948,553	1,342,115	3.253	0.000	0.051	0.075
Casanare	7	11	11	2	273,663	276,891	298,135	1,897,555	2.558	3.973	3.690	0.105
Nariño	9	0	0	0	384,686	383,005	394,874	2,045,984	2.340	0.000	0.000	0.000
Boyaca	6	0	0	0	370,345	428,324	448,895	1,222,434	1.620	0.000	0.000	0.000
Tolima	8	0	0	0	547,647	583,166	685,274	733,644	1.461	0.000	0.000	0.000
Norte De Santander	5	13	0	0	389,694	455,711	467,782	407,143	1.283	2.853	0.000	0.000
Caqueta	22	6	3	3	1,845,226	1,861,776	1,992,767	451,994	1.192	0.322	0.151	0.664
Arauca	11	1	2	3	1,048,543	1,096,641	1,162,032	1,209,520	1.049	0.091	0.172	0.248
Bolivar	4	7	3	3	748,701	837,567	1,172,503	35,304	0.534	0.836	0.256	8.498
Valle Del Cauca	2	0	0	0	459,596	460,727	523,306	538,101	0.435	0.000	0.000	0.000
Cauca	1	8	2	0	1,357,512	1,305,984	1,415,681	2,134,723	0.074	0.613	0.141	0.000
Choco	1	3	0	0	1,942,770	2,052,151	2,140,312	1,482,922	0.051	0.146	0.000	0.000
Amazonas	0	0	0	0	1264	1552	823	854	0.000	0.000	0.000	0.000
Antioquia	0	8	8	2	2,632,125	2,760,633	3,038,779	3,090,631	0.000	0.290	0.263	0.065
Atlantico	0	0	0	0	224,978	217,003	238,413	723	0.000	0.000	0.000	0.000
Caldas	0	0	0	0	1,340,049	1,486,685	1,809,702	1,181,505	0.000	0.000	0.000	0.000
Cundinamarca	0	0	0	0	26,125	33,725	34,238	2,134,681	0.000	0.000	0.000	0.000
Guainia	0	0	0	0	4703	4703	4547	1,449,508	0.000	0.000	0.000	0.000
Guaviare	0	0	0	0	281,611	301,224	406,242	5711	0.000	0.000	0.000	0.000
Huila	0	0	0	0	415,246	370,884	411,720	443,633	0.000	0.000	0.000	0.000
La Guajira	0	0	1	0	285,298	242,137	286,962	432,587	0.000	0.000	0.348	0.000
Magdalena	0	24	12	2	1,207,764	1,205,715	1,359,672	302,483	0.000	1.991	0.883	0.661
Risaralda	0	0	0	0	109,117	117,209	109,334	83,221	0.000	0.000	0.000	0.000
San Andres	0	0	0	0	825	801	735	108,447	0.000	0.000	0.000	0.000
Santander	0	0	0	0	1,412,313	1,442,936	1,595,532	1,617,398	0.000	0.000	0.000	0.000
Sucre	0	20	25	21	862,008	992,238	1,076,327	1,103,675	0.000	2.016	2.323	1.903
Vaupes	0	0	0	0	1223	1074	1159	1205	0.000	0.000	0.000	0.000
Vichada	0	5	8	5	242,633	231,684	254,820	262,311	0.000	2.158	3.139	1.906
Bogota D.C.	0	0	0	0	925,446	1,002,017	1,172,465	242,856	0.000	0.000	0.000	0.000
**Total**	**309**	**129**	**89**	**43**	**22,689,420**	**23,475,022**	**26,413,227**	**27,234,027**	**1.362**	**0.550**	**0.337**	**0.158**

## Data Availability

Data is available upon reasonable request.
